# Novel Porcine Epidemic Diarrhea Virus (PEDV) Variants with Large Deletions in the Spike (S) Gene Coexist with PEDV Strains Possessing an Intact S Gene in Domestic Pigs in Japan: A New Disease Situation

**DOI:** 10.1371/journal.pone.0170126

**Published:** 2017-01-17

**Authors:** Nguyen Van Diep, Junzo Norimine, Masuo Sueyoshi, Nguyen Thi Lan, Ryoji Yamaguchi

**Affiliations:** 1 Laboratory of Veterinary Pathology, Department of Veterinary, Graduate School of Medicine and Veterinary Medicine, University of Miyazaki, Miyazaki, Japan; 2 Department of Veterinary Pathology, Faculty of Veterinary Medicine, Vietnam National University of Agriculture, Hanoi, Vietnam; Sun Yat-Sen University, CHINA

## Abstract

Since late 2013, after an absence of seven years, outbreaks of porcine epidemic diarrhea virus (PEDV) infection have reemerged and swept rapidly across Japan, resulting in significant economic losses. In this study, we report the emergence, mixed infection, and genetic characterization of 15 novel field PEDV variants with large genomic deletions. The sizes of deletion varied between 582 nt (194 aa) and 648 nt (216 aa) at positions 28–714 (10–238) on the S gene (protein). Among 17 PEDV samples isolated from individual pigs, all of them contained at least two distinct genotypes with large genomic deletions, and 94.1% of them were found to consist of strains with an intact S gene. These variants were found in eight primary and nine recurrent outbreaks, and they might be associated with persistent PEDV infection in the farms. Full-length S and ORF3 genes of eight variants derived from 2 samples were characterized. This is the first report of mixed infections caused by various genotypes of PEDV and would be important for the studies of viral isolation, pathogenesis, and molecular epidemiology of the disease.

## Introduction

Porcine epidemic diarrhea (PED) is a highly contagious enteric disease characterized by vomiting, acute watery diarrhea, and in particular, a high mortality rate in suckling piglets, resulting in substantial economic losses [1]. The etiologic agent of this disease is the PED virus (PEDV), which is an enveloped, positive-sense, single-stranded RNA virus that belongs to the order *Nidovirales*, family *Coronaviridae*, and genus *Alphacoronavirus*. The disease was initially reported in England in 1971 [2] and Belgium in 1978 [3]. Since then, it has been identified in many swine-producing countries such as those in Europe and Asia, notably Belgium, Czech Republic, Hungary, Italy, China, South Korea, Japan, Thailand, and Taiwan [1, 4]. Between 2008 and 2010, new PEDV strains characterized by multiple insertions/deletions or mutations on the spike (S) protein compared with previous endemic strains have been reported in South Korea [5] and China [6, 7]. In April 2013, PED was identified in the US for the first time, and it rapidly spread across the country, causing the deaths of more than eight million newborn piglets during a one year-epidemic period [8, 9]. US-like PEDV strains have been reported in Germany [10], France [11], Belgium [12], South Korea [13], Taiwan [14], and Japan [15]. Currently, there are two PEDV types identified in the US: the original highly pathogenic (North American) PEDV [8] and the S INDEL PEDV, which has insertions and deletions in the N-terminal region of the S protein, with reportedly mild virulence in the field [9, 16]. Three novel PEDV strains with large deletions in the S gene have recently been identified in the US [17], South Korea [18], and Japan [19].

The PEDV genome is approximately 28 kb long, and it comprises at least seven open reading frames (ORF1a, ORF1b, and ORF2–6) that encode four structural proteins, namely S, envelope (E), membrane (M), and nucleocapsid (N); two nonstructural polyproteins (pp1a and pp1b); and one accessory protein encoded by the ORF3 gene [1]. The S gene exhibits a high degree of genetic diversity [5, 20, 21], and the S protein plays important roles in interactions with cellular receptors during virus entry, the induction of neutralizing antibodies in natural hosts, growth adaptation in vitro, and attenuation of virulence in vivo [22]. Thus, the PEDV S glycoprotein is utilized for determining genetic relatedness among PEDV isolates and developing diagnostic assays and effective vaccines [23, 24]. Apart from the S gene, ORF3 is the only accessory gene in PEDV, and its product is believed to function as an ion channel and influence virus production and virulence [25, 26]. The ORF3 gene has been used to differentiate between field and vaccine-derived isolates. Vaccine-derived isolates with unique deletions of 49, 51, and 4 nt on the ORF3 gene have been confirmed [15, 21, 27]. Hence, these deletions can be used to differentiate between field and attenuated vaccine PEDV viruses.

In Japan, PED was first reported in late 1982 and early 1983 [28], followed by pandemics between 1993 and 1996 [29, 30]. In an acute outbreak of PED in 1996, more than 39,000 suckling pigs died [30]. Thereafter, only sporadic PED cases were reported from 1997 to 2005. After an absence of seven years, PED reemerged, sweeping rapidly across the country since October 2013 [31]. Till August 2015, more than 490,000 pigs from approximately 1,000 infected farms have died of PED in Japan, according to the Ministry of Agriculture, Forestry, and Fisheries (http://www.maff.go.jp).

In this study, we report on PED outbreaks with popular prevalence and mixed infection of novel variants characterized by large deletions of the S gene in conjunction with a survey to investigate the origin and identify the heterogeneity among PEDV strains that have been circulating in Japan. We believe that our findings provide valuable insight into the causes and molecular epidemiology of the recently reemerging PED pandemic in Japan.

## Materials and Methods

### Sample collection, RNA extraction, and PEDV detection

Three hundred fifty-one intestinal and fecal samples were collected from individual pigs (suckling, weaned, and sows) from 176 pig farms (farrow-to-finish and farrow-to-wean) in six prefectures (Aomori, Hokkaido, Akita, Aichi, Miyazaki, Kagoshima) in Japan that experienced acute diarrhea between December 2013 and June 2015. Sample collection and RNA isolation were performed as previously reported [15]. PEDV infection was evaluated by reverse transcription-polymerase chain reaction (RT-PCR) using a published primer pair [15, 32].

### Ethics statement

The samples were obtained from a naturally infected animal in the field by qualified veterinarians as a part of normal veterinary care and diagnostic testing procedures. Samples of intestine used were collected from dead piglets and the fecal samples were non-invasively collected immediately after excretion. Therefore, no aggressive operation had been conducted against pigs for sampling purpose. No piglets or other animals were sacrificed for the purposes of this study. The University of Miyazaki DNA recombinant Committee approved the protocol for our cloning work of PEDV (protocol number 2014–464).

### Amplification of the partial S gene, full-length S gene, and ORF3 gene

**Fig 1:**
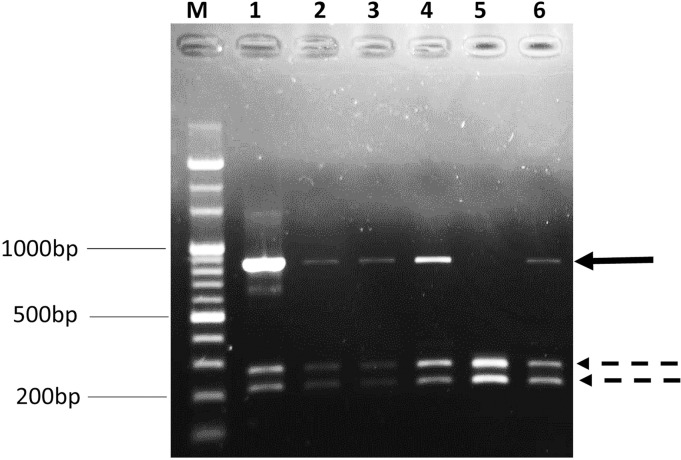
PCR product patterns amplified by the primer pair CS1-F/CS1-R targeting the partial S gene. Samples from left to right: (1) JAi-23, (2) JMi-69, (3) JMi-124, (4) JKa-230, (5) JA0-56, (6) JMi-231, (M) DNA marker. The dashed arrows indicate products of PEDV variants with large genomic deletions (approximately 0.2–0.3 kbp) and the solid arrow indicates the predicted product (872 bp).

Reverse transcription was performed using random primers and oligo-dT primers from Reverse Transcription System Kits (Promega, Madison, WI, USA). To determine the complete S gene sequence, five primer sets (CS1—CS5, Table 1) were used to amplify five DNA fragments spanning the entire S gene. These primers were designed on the basis of the published sequences of reference PEDV strains [5, 9, 33]. EmeraldAmp MAX PCR Master Mix kit (Takara Bio, Japan) containing high fidelity DNA polymerases was used for performing the PCR amplifications under the following conditions: denaturation at 94°C for 2 min; 35 cycles of denaturation at 94°C for 30 s, annealing at 54°C for 30 s and extension at 72°C for 1 min; a final extension step at 72°C for 10 min. When a partial S gene was amplified using the primer pair CS1-F/CS1-R from generated cDNA, one or two distinct bands of unexpected shorter sizes were observed in each PCR product in addition to the predicted band (Fig 1). The same result was observed when one-step RT-PCR tests (AccessQuick RT-PCR System kit, Promega Corp, WI, USA) were performed from extracted RNA and PCR tests using other kits containing high fidelity DNA polymerases (KOD FX, KOD FX neo, and Blend–Tag Plus Kits, Toyobo Co., Japan) were performed from cDNA templates to amplify the CS1 fragment. The unexpected and expected DNA bands were also observed and distinguished upon performing PCR amplifications with longer fragments containing the first portion of the S gene (primer pairs CS1-F/CS2-R, CS1-F/CS3-R, and CS1-F/CS4-R). The PCR products containing the CS1 fragment were individually gel-extracted and cloned into pCR2.1-TOPO vectors (Thermo Fisher Scientific, US). The cloning reaction was then chemically transformed into One Shot TOP10 competent cells (Thermo Fisher Scientific, US). Plasmid DNA was isolated using the FastGene Plasmid Mini Kit (Nippon Genetics, Japan). From each PEDV sample, at least 10 transformant colonies were selected for sequencing with a primer pair of the cloning vector (M13 Forward [−20] and M13 Reverse).

**Table 1:** The primers utilized in this study.

Primer name	Nucleotide sequence (5′-3′)	Target gene (fragment size, bp)	Position^*^
FS-F	TCCATTAGTGATGTTGTGTTAGG	S gene	20530–20552
CS1-F	TGCTAGTGCGTAATAATGAC	Partial S gene (872)	20573–21444
CS1-R	CATGCACCAAAGTGGAATCATT		
CS2-F	AATGGCCACATACCAGAAGG	Partial S gene (919)	21372–22290
CS2-R	GTGAAATGGTAAATTGTCTAGTGTCAA		
CS3-F	GCATCTGACACTACTATCAATGG	Partial S gene (823)	22224–23046
CS3-R	TAACAGGCGTGTTGTAAAGCTG		
CS4-F	GTATTCCCACCAACTTTAGTATGAG	Partial S gene (855)	22981–23835
CS4-R	CAATAGAACTAGAAATGGCTTGGAAG		
CS5-F	CTTACCGTACAGCTGCAACAC	Partial S gene (1058)	23787–24844
CS5-R	GACATCTTTGACAACTGTGT		
ORF3-F	GTCCTAGACTTCAACCTTACGAAG	ORF3 gene (740)	24745–25484
ORF3-R	AACTACTAGACCATTATCATTCAC		

* Numbers correspond to positions within the Colorado/USA/2013 genome.

To obtain the sequence of the full-length S gene and ORF3 gene of PEDV strains coexisting in a single PEDV sample, a fragment containing these two contiguous genes was amplified using the primer set FS-F/ORF3-R and the EmeraldAmp MAX PCR Master Mix kit (Takara Bio, Japan). The PCR conditions were as follows: denaturation at 94°C for 2 min; 35 cycles of denaturation at 94°C for 30 s, annealing at 55°C for 30 s and extension at 72°C for 5 min; a final extension step at 72°C for 10 min. The expected size of this amplified product was 4955 bp. Subsequently, the PCR products were gel-extracted and cloned into pCR-XL-TOPO plasmid vectors (TOPO XL PCR Cloning Kit, Invitrogen, US). After transformation into competent cells, plasmid DNA isolation was performed as described previously. The purified plasmid DNA was sequenced using the primer sets CS1–CS5 and the primer set ORF3-F/ORF3-R [15]. However, when the fragment containing entire S and ORF3 genes was amplified, PCR products derived from only two samples JKa-295 and JMi-277 were visualized distinctly in agarose gel 0.8%. Therefore, these two samples were selected for cloning and sequencing of the full-length S and ORF3 genes.

### Sequencing analysis

All sequences were determined in both directions, and sequencing reactions were performed using BigDye Terminator v3.1 Cycle Sequencing Kits and an ABI PRISM 3130xl Genetic Analyzer (Applied Biosystems, US). Nucleotide and deduced amino acid sequences were edited, assembled, and analyzed using Geneious v9.0.4 software (http://www.geneious.com) and molecular evolutionary genetics analysis (MEGA) software version 6.0 [34]. The resultant nucleotide sequences were deposited in GenBank under the following accession numbers: KU363044–KU363130. Phylogenetic trees based on the nucleotide sequences of the full-length S gene and ORF3 gene were constructed by the maximum likelihood method using the Tamura-Nei substitution model with a discrete gamma distribution; then gaps/missing data treatment was done by complete deletion, and bootstrap tests of 1000 replicates in the MEGA program.

## Results and Discussion

A total of 248 field samples (70.7%) collected from animals at 145 farms (82.4%) experiencing acute diarrhea in six prefectures were positive for PEDV. The result indicated that PEDV has a high prevalence rate in Japanese pig herds. Notably, PED outbreaks were commonly observed in PED-vaccinated farms, and there was a significant difference in the mortality rate (0–100%) for piglets and durations of diarrhea among PEDV-infected farms. This prompted critical questions regarding the molecular characteristics and virulence of circulating PEDV as well as efficacy of PEDV vaccines that have been used in Japan.

To monitor heterogeneity among PEDV strains, 52 PEDV-positive samples were selected for further sequence analyses. However, when CS1-F/CS1-R primers were used for PCR to amplify the first portion of the S gene, one or two distinct bands of unexpected size (approximately 0.2–0.3 kbp) were observed in each PCR product of 17 PEDV samples (32.7%) in addition to the predicted band (872 bp). The PCR products were individually gel-extracted, cloned, and sequenced.

Sequencing results illustrated that the unexpected bands were fragments with large deletions in the S gene, and a total of 15 novel PEDV genotypes with large deletions were identified (Table 2). The sizes of deletions found in this study were 582 (194 aa), 591 (197 aa), 603 (201 aa), 606 (202 aa), 609 (203 aa), 612 (204 aa), 615 (205 aa), 627 (209 aa), 633 (211 aa), 636 (212 aa), 642 (214 aa), 645 (215 aa), and 648 (216 aa) nt. These deletions occurred at positions 28–714 (10–238) in the S gene (protein). Although two genotypes feature 606-nt deletions, the deletions were located at two distinct positions (94–699 and 46–651) in the S gene. Furthermore, the genotype with a 203-aa deletion at residues 10–217 featured a 5-aa insertion (YAMFF) at the position of the deletion. Interestingly, the identical nucleotide sequence corresponding to the insertion (TACGCCATGTTCTTT) existed in the middle of the S gene (nt 2091–2105). Additionally, all PEDV variants of the S protein identified in this study were predicted to have signal peptide cleavage sites using the SignalP 4.1 server (http://www.cbs.dtu.dk/services/SignalP-4.1). This indicates that the S proteins of the variants are most likely expressed as structural proteins.

**Table 2:** Fifteen genotypes with large deletions in the S gene of the Japanese PEDV strains in this study.

No	Characteristic of the deletion				PEDV samples
	Length	Position on S gene	Position on S protein	Signal peptide cleavage sites (between positions)	
1	582 nt (194 aa)	73–654	25–218	20 and 21	JAo-56, JMi-69, JMi-124, JKa-230, JMi-231, JMi-238, JMi-277, JMi-278, JMi-283, JKa-295,JAi-312, JAi-318
2	591 nt (197 aa)	100–690	34–230	20 and 21	JMi-277, JKa-292, JKa-295,JAi-312, JAi-318
3	603 nt (201 aa)	64–666	22–222	20 and 21	JMi-235, JMi-124
4	606 nt (202 aa)	94–699	32–233	20 and 21	JAi-23, JMi-238,JMi-239, JMi-277,
5	606 nt (202 aa)	46–651	16–217	18 and 19	JMi-124
6	609 nt (203 aa)	28–651	10–217	18 and 19	JAi-29, JMi-235, JMi-239,JMi-278, JMi-283, JKa-292
7	612 nt (204 aa)	70–681	24–227	20 and 21	JAi-23, JAi-29, JMi-69
8	612 nt (204 aa)	79–690	27–230	20 and 21	JKa-292, JKa-295
9	615 nt (205 aa)	67–681	23–227	18 and 19	JKa-295
10	627 nt (209 aa)	97–723	33–241	20 and 21	JAi-23
11	633 nt (211 aa)	61–693	21–231	18 and 19	JKa-295
12	636 nt (212 aa)	85–720	29–240	20 and 21	JAo-56, JKa-292
13	642 nt (214 aa)	70–711	24–237	25 and 26	JAi-23, JAi-29, JMi-69, JKa-230, JMi-231, JMi-238, JMi-278,JMi-283, JAi-312
14	645 nt (215 aa)	67–711	23–237	23 and 24	JKa-295
15	648 nt (216 aa)	67–714	23–238	18 and 19	JAo-56, JKa-295

Surprisingly, mixed infection with multiple variants was found in all PEDV samples (17/17) collected from individual pigs in primary or recurred PED outbreaks. Particularly, in sample JKa-295, we found nine coexisting genotypes including a genotype with an intact S gene and eight genotypes with deletions of 194, 197, 201, 204, 205, 211, 215, and 216 aa (Table 3). Identical genotypes were also isolated from different farms of various prefectures that are geographically distant from each other. In addition to the variants, PEDV strains with an intact S gene were detected in 94.1% of the samples (16/17), with no intact gene detected in sample JAo-56. These results revealed that complex mixed-genotype infection of PEDV had occurred in individual pigs and farms.

**Table 3:** Sixty-seven PEDV field strains collected in Japan during outbreaks from December 2013 to June 2015.

Sample	Outbreak	Farm	Prefecture	Collected time	PEDV strains	Sequence/ deletion	S geneacce No	ORF3 geneacce No
JAi-23	Primary outbreak	ToA	Aichi	Apr-2014	JAi-23/CS1norCo1	CS1/no	KU363044	
					JAi-23/CS1de202	CS1/202 aa	KU363045	
					JAi-23/CS1de204	CS1/204 aa	KU363046	
					JAi-23/CS1de209	CS1/209 aa	KU363047	
					JAi-23/CS1de214	CS1/214 aa	KU363048	
JAi-29	Primary outbreak	ToB	Aichi	Apr-14	JAi-29/CS1nor	CS1/no	KU363049	
					JAi-29/CS1de203	CS1/203 aa	KU363050	
					JAi-29/CS1de204	CS1/204 aa	KU363051	
JAo-56	Primary outbreak	Mi	Aomori	May-14	JAo-56/CS1de194	CS1/194 aa	KU363052	
					JAo-56/CS1de212	CS1/212 aa	KU363053	
					JAo-56/CS1de216	CS1/216 aa	KU363054	
JMi-69	Primary outbreak	Na	Miyazaki	Apr-14	JMi-69/CS1nor	CS1/no	KU363055	
					JMi-69/CS1de194	CS1/194 aa	KU363056	
					JMi-69/CS1de204	CS1/204 aa	KU363057	
					JMi-69/CS1de214	CS1/214 aa	KU363058	
JMi-124	Primary outbreak	Ok	Miyazaki	Mar-14	JMi-124/CS1nor	CS1/no	KU363059	
					JMi-124/CS1de194	CS1/194 aa	KU363060	
					JMi-124/CS1de201	CS1/201 aa	KU363061	
					JMi-124/CS1de202	CS1/202 aa	KU363062	
JKa-230	Primary outbreak	Kac1	Kagoshima	Jul-14	JKa-230/CS1nor	CS1/no	KU363063	
					JKa-230/CS1de194	CS1/194 aa	KU363064	
					JKa-230/CS1de214	CS1/214 aa	KU363065	
JMi-231†	Second outbreak	Ho	Miyazaki	Mar-2014	JMi-231/CS1nor	CS1/no	KU363066	
					JMi-231/CS1de194	CS1/194 aa	KU363067	
					JMi-231/CS1de214	CS1/214 aa	KU363068	
JMi-235†	Third outbreak	Ho	Miyazaki	Jun-14	JMi-235/CS1nor	CS1/no	KU363069	
					JMi-235/CS1de201	CS1/201 aa	KU363070	
					JMi-235/CS1de203	CS1/203 aa	KU363071	
JMi-238	Second outbreak	Te	Miyazaki	Jun-14	JMi-238/CS1nor	CS1/no	KU363072	
					JMi-238/CS1de194	CS1/194 aa	KU363073	
					JMi-238/CS1de202	CS1/202 aa	KU363074	
					JMi-238/CS1de214	CS1/214 aa	KU363075	
JMi-239	Second outbreak	Sa	Miyazaki	Jul-14	JMi-239/CS1nor	CS1/no	KU363076	
					JMi-239/CS1de202	CS1/202 aa	KU363077	
					JMi-239/CS1de203	CS1/203 aa	KU363078	
JMi-277	Second outbreak	No	Miyazaki	May-14	JMi-277/CS1nor	CS1/no	KU363079	
					JMi-277/CS1de194	CS1/194 aa	KU363080	
					JMi-277/CS1de197	CS1/197 aa	KU363081	
					JMi-277/CS1de202	CS1/202 aa	KU363082	
					JMi-277fSnorCo11	Full S/no	KU363111	KU363122
					JMi-277fSnorCo12	Full S/no	KU363112	KU363123
					JMi-277fSnorCo13	Full S/no	KU363113	KU363124
					JMi-277fSde197Co26	Full S/197 aa	KU363114	KU363125
					JMi-277fSde197Co27	Full S/197 aa	KU363115	KU363126
					JMi-277fSde197Co28	Full S/197 aa	KU363116	KU363127
JMi-278	Second outbreak	Is	Miyazaki	Feb-14	JMi-278/CS1nor	CS1/no	KU363083	
					JMi-278/CS1del194	CS1/194 aa	KU363084	
					JMi-278/CS1del203	CS1/203 aa	KU363085	
					JMi-278/CS1de214	CS1/214 aa	KU363086	
JMi-283	Second outbreak	Oi	Miyazaki	Jun-14	JMi-283/CS1nor	CS1/no	KU363087	
					JMi-283/CS1de194	CS1/194 aa	KU363088	
					JMi-283/CS1de203	CS1/203 aa	KU363089	
					JMi-283/CS1de214	CS1/214 aa	KU363090	
JKa-292	Primary outbreak	Kap2	Kagoshima	December-13	JMi-292/CS1nor	CS1/no	KU363091	
					JMi-292/CS1de197	CS1/197 aa	KU363092	
					JMi-292/CS1de203	CS1/203 aa	KU363093	
					JMi-292/CS1de204	CS1/204 aa	KU363094	
					JKa-292/CS1de212	CS1/212 aa	KU363095	
JKa-295	Primary outbreak	Kap5	Kagoshima	Jan-14	JMi-295/CS1nor	CS1/no	KU363096	
					JKa-295/CS1de194	CS1/194 aa	KU363097	
					JKa-295/CS1de197	CS1/197 aa	KU363098	
					JKa-295/CS1de201	CS1/201 aa	KU363099	
					JKa-295/CS1de204	CS1/204 aa	KU363100	
					JKa-295/CS1de205	CS1/205 aa	KU363101	
					JKa-295/CS1de211	CS1/211 aa	KU363102	
					JKa-295/CS1de216	CS1/216 aa	KU363103	
					JKa-295fSde194Co25	Full S/194 aa	KU363121	KU363132
					JKa-295fSde197Co4	Full S/197 aa	KU363117	KU363128
					JKa-295fSde197Co5	Full S/197 aa	KU363118	KU363129
					JKa-295fSde197Co8	Full S/ 197 aa	KU363120	KU363131
					JKa-295fSde215Co6	Full S/ 215 aa	KU363119	KU363130
JAi-312	Second outbreak	Tom	Aichi	Jan-15	JAi-312/CS1nor	CS1/ no	KU363104	
					JAi-312/CS1de194	CS1/194 aa	KU363105	
					JAi-312/CS1de197	CS1/197 aa	KU363106	
					JAi-312/CS1de214	CS1/214 aa	KU363107	
JAi-318	Third outbreak	At	Aichi	Jun-15	JAi-318/CS1nor	CS1/no	KU363108	
					JAi-318/CS1de194	CS1/194 aa	KU363109	
					JAi-318/CS1de197	CS1/204 aa	KU363110	

†: PEDV samples with the same the symbol were collected from the same farm.

S gene acce No: GenBank accession number of the S sequence.

ORF3 gene acce No: GenBank accession number of the ORF3 sequence.

Note: The sequence of the S gene could be partial (CS1 fragment) or whole. Eleven strains with sequences of full-length S genes that were obtained, including JMi-277fSnorCo11, JMi-277fSnorCo12, JMi-277fSnorCo13, JMi-277fSde197Co26, JMi-277fSde197Co27, JMi-277fSde197Co28, JKa-295fSde197Co4, JKa-295fSde197Co5, JKa-295fSde215Co6, JKa-295fSde197Co8, and JKa-295fSde194Co25. Each sequence representing a distinct PEDV genotype within a field sample was isolated from an individual colony.

Full-length S and ORF3 genes of PEDV strains derived from samples JKa-295 and JMi-277 were cloned and sequenced. Identical nucleotide sequences of both S and ORF3 genes were distinguished and excluded, resulting in the identification of 11 individual strains (Table 3). Three strains that were obtained had an intact S gene (4161 nt, 1386 aa), whereas eight other variants possessed deletions of 582 (194 aa), 591 (197 aa), and 645 (215 aa) nt. Notably, the variants with a 197-aa deletion were identical to that of US strain PC177-P2, which was proposed to be obtained during adaptation of the virus in cell culture [17]. In addition, the 194-aa deletion from the variants in this study was similar to that of the Tottori2 strain that has been recently isolated from domestic pigs in Japan [19]. However, apart from Tottori2, all variants with a 194-aa deletion in our study were found to coexist with other variants as well as strains with an intact S gene.

The PEDV S protein is a type I glycoprotein [35]. Its identified epitope regions include the COE domain (499–638 aa) and epitopes SS2 (748–755 aa), SS6 (764–771 aa), and 2C10 (1368–1374 aa) [36–38]. It was demonstrated that the large deletions found in the S gene of the variants did not occur in these four regions, and there was no significant difference in the epitope regions between the PEDV variants and PEDV strains with an intact S gene (S1 Fig).

**Fig 2:**
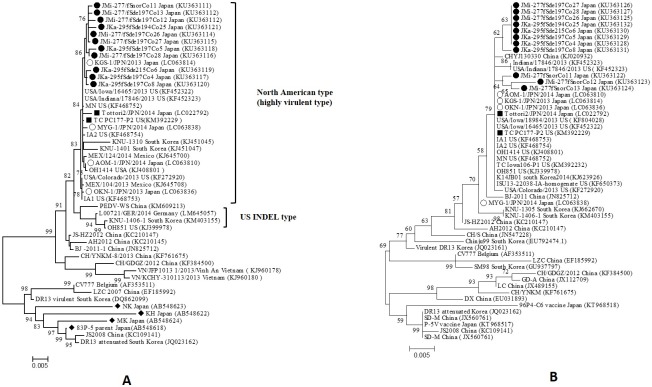
**Phylogenetic tree based on the nucleotide sequences of the entire S gene (A) and ORF3 gene (B) of 11 PEDV strains in this study and other reference strains identified worldwide**. The tree was constructed by the maximum likelihood method using the Tamura-Nei substitution model with a discrete gamma distribution in the MEGA v.6.05 program. Numbers at nodes represent the percentage of 1000 bootstrap replicates. The scale bar indicates the number of nucleotide substitutions per site. The 11 PEDV strains evaluated in this study are marked by solid round symbols, other PEDV strains recently isolated in Japan are marked by hollow round symbols, the Japanese strains identified prior to 2013 are marked by solid diamond symbols, and two known strains possessing large deletions in the S gene, Tottori2/JPN/2014 and TC PC177-P2, are marked by solid rectangle symbols.

Sequencing analysis indicated that strains found in a single PEDV sample displayed genetic diversity in their S genes. In sample JMi-277, three strains with an intact S gene had 99.64–99.78% nucleotide sequence identity with each other and 99.64–99.89% nucleotide sequence identity with three variants possessing the 197-aa deletion when pairwise distances were measured using MEGA v6.05 software. Within JKa-295, three variants with the 197-aa deletion shared 99.72–99.92% nucleotide sequence identity with each other but had 99.38–99.55% nucleotide sequence identity with JKa-295fSde204Co25, which possessed the 204-aa deletion (S1 Table). On the contrary, all strains found in JKa-295 and JMi-277 had the greatest nucleotide sequence identity (98.71–99.92%) with highly virulent strains isolated recently in the US, South Korea, and Japan [9, 13, 31] but exhibited relatively less nucleotide sequence identity (93.34–95.99%) with Japanese PEDV strains (NK, MK, KH, and 83P-5) reported prior to 2013 [39]. Furthermore, phylogenetic analysis based on the full-length S gene classified these Japanese strains into the North American (highly virulent) type as shown in Fig 2A. It is suggested that the variants originated from North American type of PEDV.

The sequencing results illustrated that the ORF3 gene of the 11 strains in JKa-295 and JMi-277 had the same length of 675 nt, and it did not possess any deletion found in known PEDV vaccine strains [15, 27, 40]. All of the variants with large deletions have the same ORF3 nucleotide sequence excluding JKa-295fSde194Co25. Nucleotide identity of the ORF3 gene between the variants and intact S strains was 98.81–99.41% which was lower than the 28 field isolates (99.6–100%) collected from 5 prefectures in Japan during 2103–2104 [15]. Eight distinct SNPs between the variants and the strains with an intact S gene were also identified at positions 136, 154, 178, 228, 302, 561, 645, and 657 in the ORF3 gene (S2 Fig). Phylogenetic analysis revealed that the strains with an intact S gene and the variants fell into two distinct clades (Fig 2B). The result indicated that the variants were not vaccine-related, and might have evolved independently as compared to the intact S strains which coexisted in the individual samples.

In this study, variants with large deletions in the S gene were found in eight primary and nine recurrent outbreaks from 16 pig farms, and they mostly (94.1%) coexisted with PEDV strains with an intact S gene. In four farms (Te, Sa, Is, and At), the variants were not found in the primary outbreaks, but they appeared in subsequent pandemics. This indicated that PEDV variants could be transmitted from farm to farm and/or that the proportion of the variants in samples collected at the primary outbreaks was insufficient to permit detection of the variants by PCR. Additionally, PED outbreaks in Japan since late 2013 were proposed to be caused by the invasion of recent overseas strains [15, 31]. Taken together, it is possible that in the recent pandemic, both the PEDV variants and the strains with an intact S gene were introduced together from other countries, after which they spread across Japan. However, the earliest PED outbreak of the variants possessing the large S deletions occurred on Kagoshima on December 2013 (sample JKa-292) while the first PED case in Japan was reported earlier in Okinawa on October 2013. Presently, no PEDVs possessing the large deletions in the N-terminus of the S gene analogous to the Japanese variants was reported in other countries except PC177-P2 which was concluded to derive from cell adaptation in vitro. Therefore, the variants might have arisen spontaneously during the PEDV spreading in Japan. Further studies are required for the confirmation.

In this study, due to the coexistence of the variants and PEDV strains with an intact S gene in all of the samples excluding JAo-56, it might not be accurate to evaluate the virulence of individual strains based solely on the information from clinical signs and mortality rates recorded from the pig farms. In JAo-56, we could not identify any PEDV strain with an intact S gene. In the farm from which this sample was collected, the sows had never been vaccinated with modified live PEDV. Piglets in the farm manifested various severities of diarrhea from mild to severe, and the mortality rate reached 72.4% (670/926). The mortality rate was much higher than the mortality rate recorded in the epidemic during which Tottori2 was isolated (0%) [19]. Therefore, the large deletion in the variants observed in this study might not necessarily lower the virulence of this virus. Further investigations are needed to elucidate the virulence of variants in PED outbreaks.

After the primary outbreaks occurred in 2013 and the first half of 2014, there was a sharp decrease of PED cases that were reported during the second half of 2014 and 2015 in Japan. Among the few recurrent PED outbreaks reported in 2014 and 2015, all the samples collected from pig farms in Miyazaki (6 farms) and Aichi (2 farms) were found to contain the PEDV variants. In those farms, diarrhea in piglets was observed for a long time (up to three months), and outbreaks of PED were reported to frequently recur in intervals from three weeks to several months (S2 Table). Although many strict measures for establishing a high level of security were implemented such as quarantine of reposition, strict visitor policies, banning of unwashed vehicles, controlling of carcasses, movement of the caretakers on the farms and using virucidal disinfectants; PED still recurred in the farms. Therefore, it is suggested that mixed infection of PEDV variants with large genomic deletions may play an important role in the persistence of PEDV in pig farms.

Defective interfering (DI) particles are a common observation in many virus families [41]. DI particles are defective for replication in the absence of the product of the deleted gene, and its replication requires the presence of the complete functional virus particle to co-infect a cell with it, in order to provide the lost factors [42]. The PEDV variants wherein large genomic deletions were observed may be DI particles. However, we speculated otherwise because the reported PEDV strains PC177-P2 and Tottori2 possessing the same deletions found in the variants of this study could replicate well in cell culture and cause cytopathic effect [17, 19]. Besides, the mutant strain PC177-P2 can propagate well and induce diarrhea in piglets [43]. A deletion of 215 aa (position 19–233) in the N-terminal domain of the S protein analogous to the deletion observed in the variants revealed a not impaired propagation of PEDV in vivo [44]. Moreover, only variants with large S deletions were found in the field sample JAo-56, which indicated that the variants were able to independently propagate well in pigs. Additionally, all the deletions found in the variants are in-frame and the S proteins were predicted to express as a functional structure protein. Taken together, it is suggested that the variants with the large S deletions in this study were not DI particles.

Functions of the spike protein N-terminal domain of PEDV and other *alphacoronaviruses* were previously elucidated [44]. The loss of this domain occurred in a number of *alphacoronaviruses* correlates with a loss or reduction of enteric tropism. The most obvious example of this phenomenon is transmissible gastroenteritis virus (TGEV) that has a dual tropism targeting the respiratory and gastrointestinal tract of the pig. Meanwhile, porcine respiratory coronavirus (PRCV) is a natural variant of TGEV which lacks the S protein N-domain. It has reduced considerably its enteric tropism and primarily replicated in the respiratory tract then, produced an attenuated disease in comparison with parental TGEV strains. Another typical example is feline coronaviruses that have 2 pathotypes: feline enteric coronavirus (FECV) causing a clinical mild or asymptomatic disease and feline infectious peritonitis (FIPV) inducing a highly pathologic disease. FIPV is thought to be derived from FECV by natural mutation. However, compared to FECV, PIPV has a large deletion in the S protein N-domain which results in the loss of tropism for enterocytes [44, 45]. These examples revealed an important role of *alphacoronavirus* spike N-domain for viral replication in the enteric tract. The N-terminus (residues 1–249) of the S protein was demonstrated to be responsible for the sialic acid binding activity of PEDV [44]. The loss of enteric tropism observed in TGEV was specifically associated with the loss of the sialic binding activity that located in the N-domain. However, how the sialic acid binding activity affects the tropism of these *alphacoronaviruses* remains unclear. The S protein N-domain of some PEDV strains were thought to be dispensable for replication of PEDV *in vitro* [44–46]. The large deletions in the S protein of the PEDV variants in this study indicated that this domain might also be dispensable *in vivo*. In the present study, the characteristics of large deletions found in the Japanese variants were highly similar to those observed in the PRCV that have a 224 or 227-aa deletion in the N-terminal domain of the S protein [47]. PRCV can persist in pig herds throughout the entire year, or it can disappear temporarily from a herd for several months and reappear subsequently. The emergence and widespread prevalence of PRCV lessened the clinical impact of TGE in the United States and Europe due to cross-protective immunity with TGEV [48]. Thus, the PEDV variants identified in this study might have biological characteristics analogous to PRCV and may persist in the pig farms of Japan. Unfortunately, we did not collect lung tissues from PEDV-infected pigs in these outbreaks, and virus isolation for the individual variants has not yet been completed. Therefore, it remains unclear whether the PEDV variants found in this study exhibited altered virulence, pathogenicity, and tissue tropism from the strains with an intact S gene. In future studies, we will continuously seek to answer this question.

## Conclusions

This is the first report describing PED outbreaks with mixed infections of various genotypes. Our study identified 15 field PEDV genotypes with large deletions in the S gene that have been circulating in Japan from 2013 to 2015. The full-length S gene and ORF3 gene of eight variants and three strains with an intact S gene were characterized. The variants coexisted with intact S strains in high frequency and they might be associated with persistent PEDV infection in pig farms in Japan. Such a reemerging and mixed infection of the variants in this study have put PED in a new situation indicating this disease has become more complex in terms of viral isolation, pathogenesis, and epidemiology. The mechanism by which the PEDV variants were generated and evolved remains unclear. Hence, further studies are required to elucidate biological properties such as changes in tissue tropism and the virulence patterns of the new variants.

## Supporting Information

S1 Fig Amino acid sequence alignments of entire S proteins from the 11 PEDV strains obtained in this study and reference strains. (PDF)

S2 Fig Amino acid sequence alignments of the ORF3 gene from the 11 PEDV strains obtained in this study. (PDF)

S1 Table Nucleotide sequence identity based on the full-length spike genes of 11 Japanese PEDV field strains identified in this study and PEDV reference strains*. (DOCX)

S2 Table Status of pig farms where the PEDV samples using in this study were collected. (DOCX)
