# Supplementary material for: Novel Porcine Epidemic Diarrhea Virus (PEDV) Variants with Large Deletions in the Spike (S) Gene Coexist with PEDV Strains Possessing an Intact S Gene in Domestic Pigs in Japan: A New Disease Situation
Source: PLoS One. 2017 Jan 17;12(1):e0170126. doi: 10.1371/journal.pone.0170126 (PMC5241010; doi:10.1371/journal.pone.0170126)
Supplement: S1 Fig — (PDF) [file pone.0170126.s001.pdf]

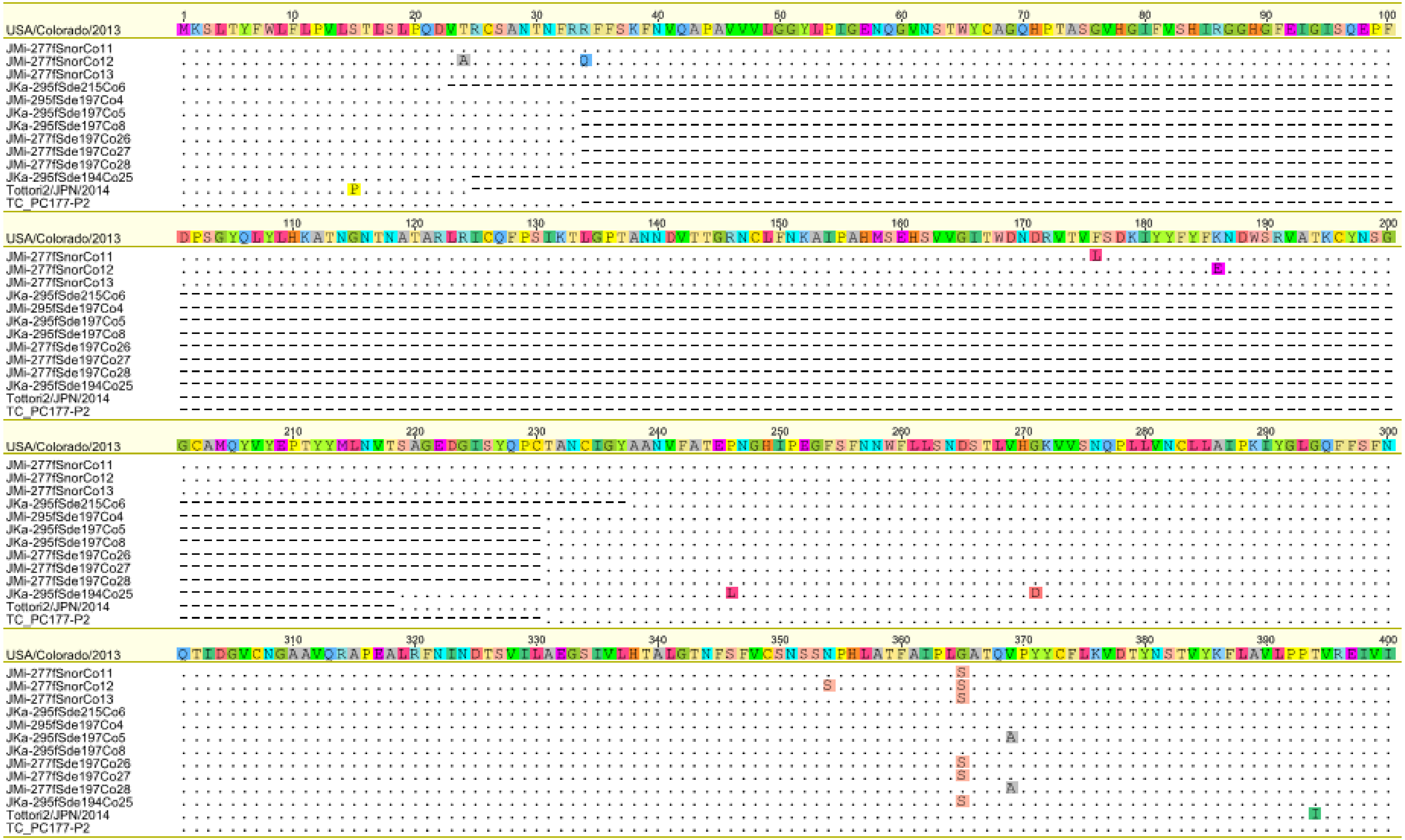
USA/Colorado/2013
JMi-277fSnorCo11
JMi-277fSnorCo12
JMi-277fSnorCo13
JKa-295fSde215Co6
JMi-295fSde197Co4
JKa-295fSde197Co5
JKa-295fSde197Co8
JMi-277fSde197Co26
JMi-277fSde197Co27
JMi-277fSde197Co28
JKa-295fSde194Co25
Tottori2/JPN/2014
TC_PC177-P2

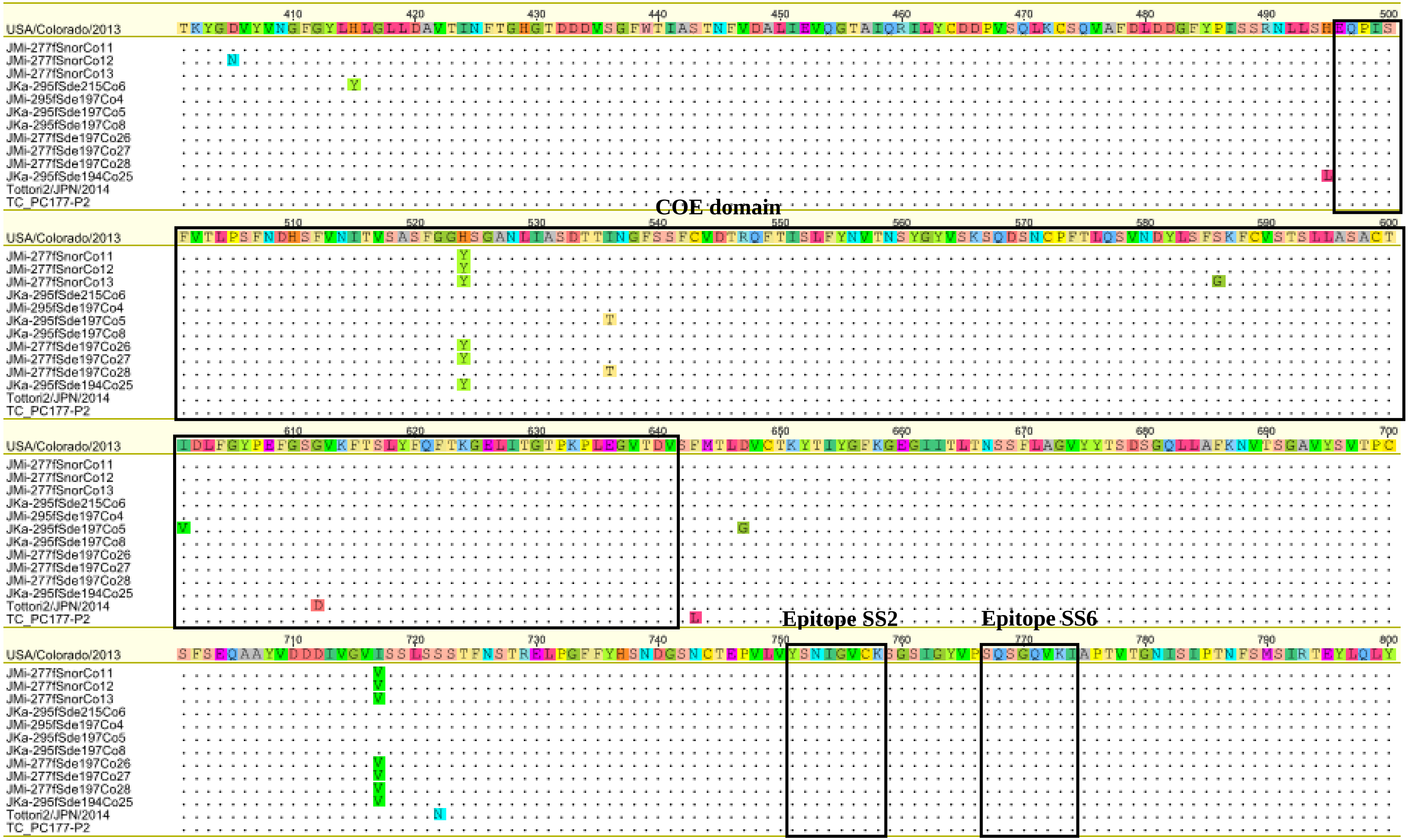
COE domain
Epitope SS2
Epitope SS6
USA/Colorado/2013
JMi-277fSnorCo11
JMi-277fSnorCo12
JMi-277fSnorCo13
JKa-295fSde215Co6
JMi-295fSde197Co4
JKa-295fSde197Co5
JKa-295fSde197Co8
JMi-277fSde197Co26
JMi-277fSde197Co27
JMi-277fSde197Co28
JKa-295fSde194Co25
Tottori2/JPN/2014
TC_PC177-P2

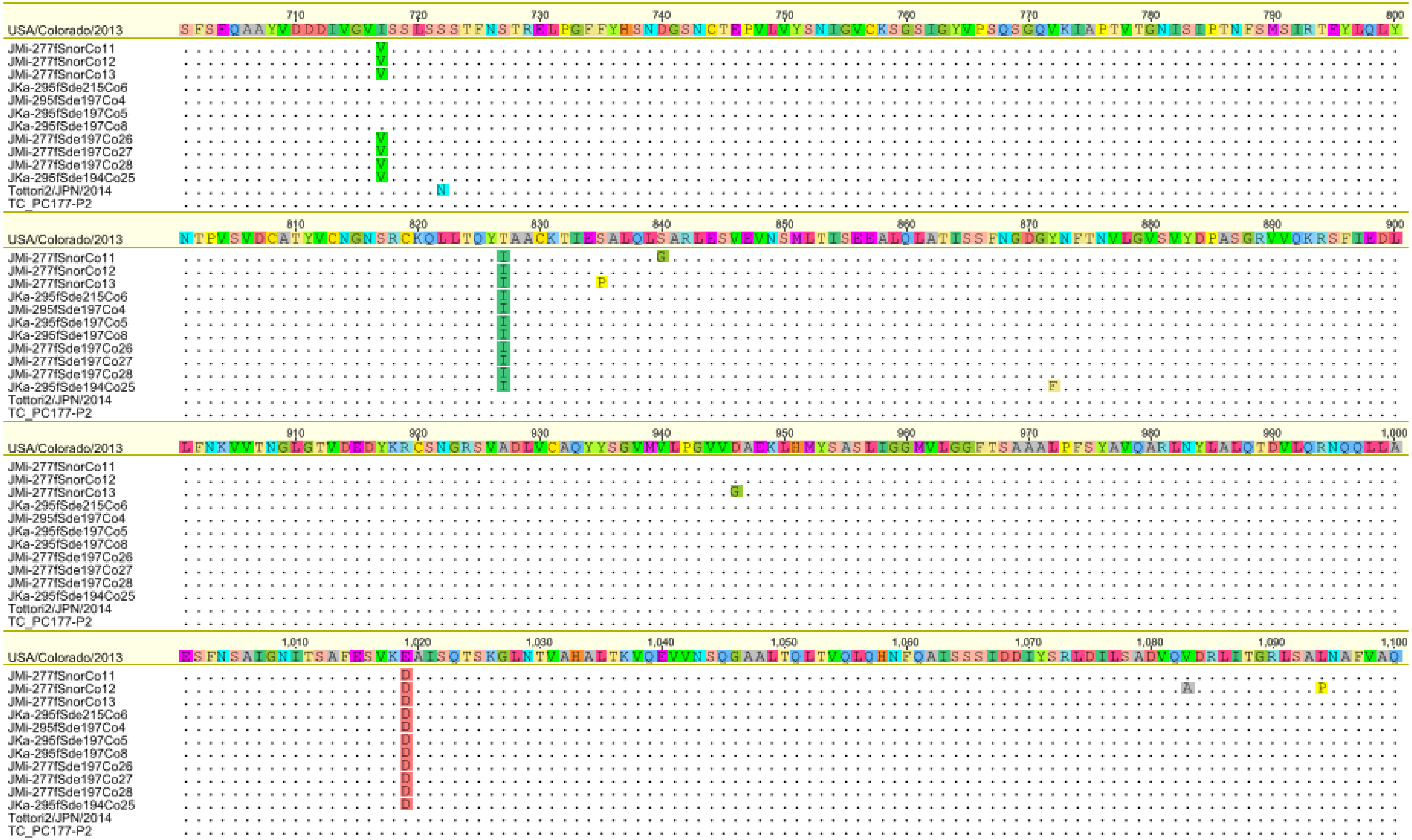
USA/Colorado/2013
JMi-277fSnorCo11
JMi-277fSnorCo12
JMi-277fSnorCo13
JKa-295fSde215Co6
JMi-295fSde197Co4
JKa-295fSde197Co5
JKa-295fSde197Co8
JMi-277fSde197Co26
JMi-277fSde197Co27
JMi-277fSde197Co28
JKa-295fSde194Co25
Tottori2/JPN/2014
TC_PC177-P2

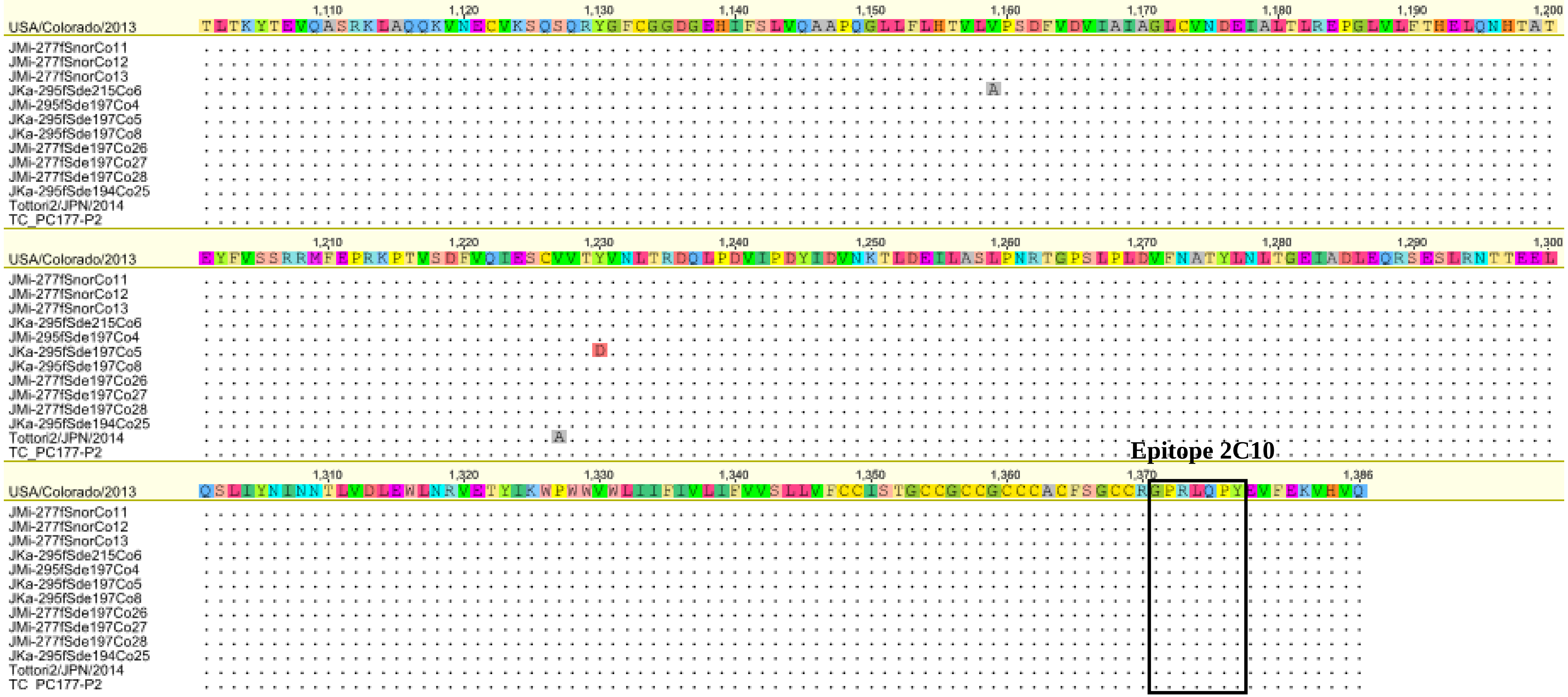
USA/Colorado/2013
JMi-277fSnorCo11
JMi-277fSnorCo12
JMi-277fSnorCo13
JKa-295fSde215Co6
JMi-295fSde197Co4
JKa-295fSde197Co5
JKa-295fSde197Co8
JMi-277fSde197Co26
JMi-277fSde197Co27
JMi-277fSde197Co28
JKa-295fSde194Co25
Tottori2/JPN/2014
TC_PC177-P2
Epitope 2C10
