# Supplementary material for: Novel Porcine Epidemic Diarrhea Virus (PEDV) Variants with Large Deletions in the Spike (S) Gene Coexist with PEDV Strains Possessing an Intact S Gene in Domestic Pigs in Japan: A New Disease Situation
Source: PLoS One. 2017 Jan 17;12(1):e0170126. doi: 10.1371/journal.pone.0170126 (PMC5241010; doi:10.1371/journal.pone.0170126)
Supplement: S2 Fig — (PDF) [file pone.0170126.s002.pdf]

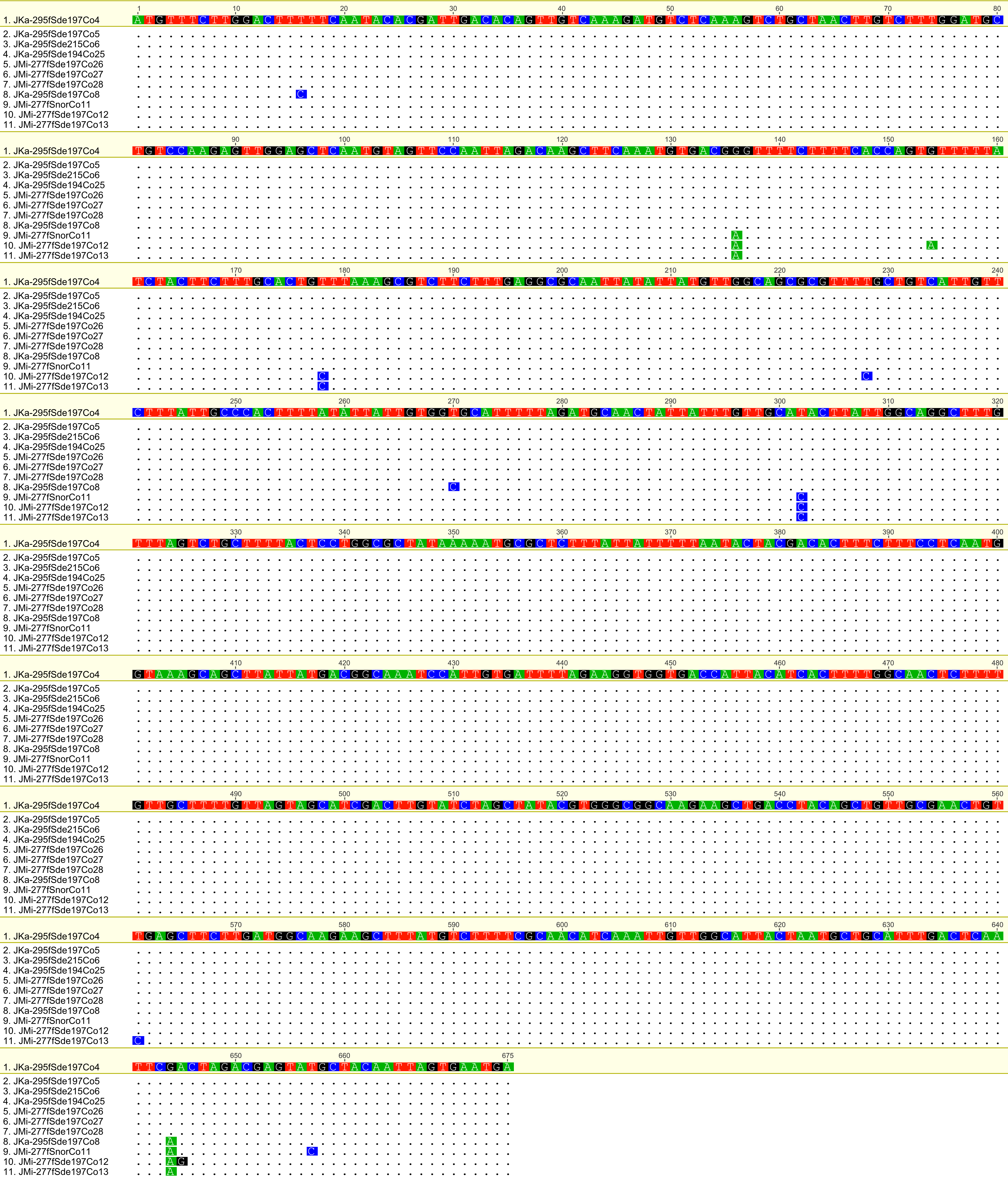
1. JKa-295fSde197Co4
2. JKa-295fSde197Co5
3. JKa-295fSde215Co6
4. JKa-295fSde194Co25
5. JMi-277fSde197Co26
6. JMi-277fSde197Co27
7. JMi-277fSde197Co28
8. JKa-295fSde197Co8
9. JMi-277fSnorCo11
10. JMi-277fSde197Co12
11. JMi-277fSde197Co13
