# Supplementary material for: Novel Porcine Epidemic Diarrhea Virus (PEDV) Variants with Large Deletions in the Spike (S) Gene Coexist with PEDV Strains Possessing an Intact S Gene in Domestic Pigs in Japan: A New Disease Situation
Source: PLoS One. 2017 Jan 17;12(1):e0170126. doi: 10.1371/journal.pone.0170126 (PMC5241010; doi:10.1371/journal.pone.0170126)
Supplement: S1 Table — (DOCX) [file pone.0170126.s003.docx]

Table S1: Nucleotide sequence identity based on the full-length spike genes of 11 Japanese PEDV field strains identified in this study and PEDV reference strains*

| Virus strain | USA/Colorado/2013 | JMi-277/fSnorCo11 | JMi-277/fSnorCo12 | JMi-277/fSnorCo13 | JKa-295/fSde215Co6 | JMi-295fSde197Co4 | JKa-295/fSde197Co5 | JKa-295/fSde197Co8 | JMi-277/fSde197Co26 | JMi-277/fSde197Co27 | JMi-277/fSde197Co28 | JKa-295fSde194Co25 | Tottori2/JPN/2014 | TC_PC177-P2 |
| --- | --- | --- | --- | --- | --- | --- | --- | --- | --- | --- | --- | --- | --- | --- |
| USA/Colorado/2013 | --- | 16 | 21 | 15 | 13 | 9 | 16 | 10 | 10 | 12 | 11 | 19 | 14 | 5 |
| JMi-277fSnorCo11 | 99.62 | --- | 15 | 9 | 15 | 11 | 18 | 12 | 4 | 6 | 9 | 13 | 20 | 11 |
| JMi-277fSnorCo12 | 99.50 | 99.64 | --- | 14 | 18 | 15 | 22 | 16 | 8 | 10 | 13 | 17 | 24 | 15 |
| JMi-277fSnorCo13 | 99.64 | 99.78 | 99.66 | --- | 15 | 11 | 18 | 12 | 4 | 6 | 9 | 13 | 20 | 11 |
| JKa-295fSde215Co6 | 99.63 | 99.57 | 99.49 | 99.57 | --- | 6 | 12 | 6 | 11 | 11 | 12 | 18 | 17 | 10 |
| JMi-295fSde197Co4 | 99.75 | 99.69 | 99.58 | 99.69 | 99.83 | --- | 7 | 3 | 7 | 5 | 8 | 16 | 15 | 6 |
| JKa-295fSde197Co5 | 99.55 | 99.50 | 99.38 | 99.50 | 99.66 | 99.80 | --- | 10 | 14 | 12 | 9 | 22 | 21 | 13 |
| JKa-295fSde197Co8 | 99.72 | 99.66 | 99.55 | 99.66 | 99.83 | 99.92 | 99.72 | --- | 8 | 8 | 9 | 17 | 16 | 7 |
| JMi-277fSde197Co26 | 99.72 | 99.89 | 99.78 | 99.89 | 99.69 | 99.80 | 99.61 | 99.78 | --- | 2 | 5 | 9 | 16 | 7 |
| JMi-277fSde197Co27 | 99.66 | 99.83 | 99.72 | 99.83 | 99.69 | 99.86 | 99.66 | 99.78 | 99.94 | --- | 7 | 11 | 18 | 9 |
| JMi-277fSde197Co28 | 99.69 | 99.75 | 99.64 | 99.75 | 99.66 | 99.78 | 99.75 | 99.75 | 99.86 | 99.80 | --- | 14 | 17 | 8 |
| JKa-295fSde194Co25 | 99.47 | 99.64 | 99.53 | 99.64 | 99.49 | 99.55 | 99.38 | 99.52 | 99.75 | 99.69 | 99.61 | --- | 21 | 16 |
| Tottori2/JPN/2014 | 99.61 | 99.44 | 99.33 | 99.44 | 99.52 | 99.58 | 99.41 | 99.55 | 99.55 | 99.49 | 99.52 | 99.41 | --- | 11 |
| TC_PC177-P2 | 99.86 | 99.69 | 99.58 | 99.69 | 99.72 | 99.83 | 99.64 | 99.80 | 99.80 | 99.75 | 99.78 | 99.55 | 99.69 | --- |

*: Three PEDV reference strains included Colorado/USA/2013 (North American type), PC177-P2 (197-aa deletion), and Tottori2 (197-aa deletion). Nucleotide identity (%) is presented in the lower triangle of table, and the numbers of different nucleotide are presented in the upper triangle of the table. Pairwise distances were evaluated using MEGA v.6.05 software, and the gaps/missing data were treated as pairwise deletion.
