# Supplementary material for: Novel Porcine Epidemic Diarrhea Virus (PEDV) Variants with Large Deletions in the Spike (S) Gene Coexist with PEDV Strains Possessing an Intact S Gene in Domestic Pigs in Japan: A New Disease Situation
Source: PLoS One. 2017 Jan 17;12(1):e0170126. doi: 10.1371/journal.pone.0170126 (PMC5241010; doi:10.1371/journal.pone.0170126)
Supplement: S2 Table — (DOCX) [file pone.0170126.s004.docx]

Table S2. Status of pif farms where the PEDV samples using in this study were collected.

| No | Samples |  | Farm | Location | Starting time of the outbreaks in the farms | | | Duration* |
| --- | --- | --- | --- | --- | --- | --- | --- | --- |
|  |  | Sample origin |  |  | 1st outbreak | 2nd outbreaks | 3rd outbreak |  |
| 1 | JAi-23 | Small Intestine | ToA | Aichi | **18.Apr.2014** |  |  | 2.5 months |
| 2 | JAi-29 | Feces | ToB | Aichi | **28.Apr.2014** | Mar.2015 |  | 3 months |
| 3 | JAo-56 | Feces | Mi | Aomori | **May.2014** |  |  | 2.5 months |
| 4 | JMi-69 | Feces | Na | Miyazaki | **20.Apr.2014** |  |  | 1 month |
| 5 | JMi-124 | Feces | Ok | Miyazaki | **Mar.2014** |  |  | ─ |
| 6 | JKa-230 | Small intestine | Kac1 | Kagoshima | **Jul.2014** |  |  | 2 months |
| 7 | JMi-231 | Feces | Ho | Miyazaki | 15.Feb.2014 | **14.Mar.2014** |  | 1 month |
| 8 | JMi-235 | Feces |  |  |  |  | **10.Jun.2014** | 2 month |
| 9 | JMi-238 | Feces | Te | Miyazaki | 13.Dec.2013 | **17.Jun.2014** |  | 1 month |
| 10 | JMi-239 | Feces | Sa | Miyazaki | 26.Mar.2014 | **04.Jul.2014** |  | 1.5 months |
| 11 | JMi-277 | Feces | No | Miyazaki | 05.Feb.2014 | **21.May.2014** |  | 3 months |
| 12 | JMi-278 | Feces | Is | Miyazaki | 08.Jan.2014 | **24.Feb.2014** |  | 2 months |
| 13 | JMi-283 | Feces | Oi | Miyazaki | 02.Jun.2014 | **27.Jun.2015** |  | 3 weeks |
| 14 | JKa-292 | Feces | Kap2 | Kagoshima | **24.Dec.2013** |  |  | 5 days |
| 15 | JKa-295 | Feces | Kap5 | Kagoshima | **09.Jan.2014** |  |  | 3 weeks |
| 16 | JAi-312 | Feces | Tom | Aichi | 02.Apr.2014 | **19.Jan.2015** |  | 1.5 months |
| 17 | JAi-318 | Feces | At | Aichi | 08.Apr.2014 | 04.Dec.2014 | **01.Jun.2015** | > 1 month **†** |

Note: The outbreaks that the PEDV samples were collected and sequenced in this study were marked in bold. The presence of PEDV in the farms was confirmed by RT-PCR using specific primers.

* Durations of diarrhea manifestation in pigs of the PED outbreaks (marked in bold) in which the variants were identified.

**†**: all the pigs in this farm were changed after approximately one month since starting of the third PED outbreak.

─: No information recorded.
